# Biomaterial-based cell delivery strategies to promote liver regeneration

**DOI:** 10.1186/s40824-021-00206-w

**Published:** 2021-02-25

**Authors:** Maqsood Ali, Samantha L. Payne

**Affiliations:** 1 Department of Regenerative Medicine, College of Medicine, Soonchunhyang University, Cheonan, South Korea; 2 Department of Biomedical Engineering, School of Engineering, Tufts University, Medford, MA 02155 USA

## Abstract

Chronic liver disease and cirrhosis is a widespread and untreatable condition that leads to lifelong impairment and eventual death. The scarcity of liver transplantation options requires the development of new strategies to attenuate disease progression and reestablish liver function by promoting regeneration. Biomaterials are becoming an increasingly promising option to both culture and deliver cells to support in vivo viability and long-term function. There is a wide variety of both natural and synthetic biomaterials that are becoming established as delivery vehicles with their own unique advantages and disadvantages for liver regeneration. We review the latest developments in cell transplantation strategies to promote liver regeneration, with a focus on the use of both natural and synthetic biomaterials for cell culture and delivery. We conclude that future work will need to refine the use of these biomaterials and combine them with novel strategies that recapitulate liver organization and function in order to translate this strategy to clinical use.

## Liver cirrhosis

Normal liver function can be disrupted by many different diseases and injury, such as viruses, drugs, poisons, carcinomas, hemochromatosis, and Wilson disease, resulting in a fibrotic response and eventual cirrhosis [1]. The liver responds to insult in one of two ways, depending on if the damage is acute or chronic. Acute liver injury is primarily caused by viruses and drugs. Although the liver is capable of regeneration in response to acute damage, prolonged insult can result in permanent tissue damage and functional impairment, eventually leading to end-stage liver disease and cirrhosis [1–4]. Cirrhosis of the liver is the end point of chronic liver fibrosis. Worldwide, cirrhosis is the 14th most common cause of death, and the one-year mortality rate is 57% for end-stage liver fibrosis [5]. From 2004 to 2010, hepatic cirrhosis accounted for more than one million deaths, which is 2% of deaths worldwide, with an additional 31 million patients living with disabilities [6]. As liver disease progresses, the costs associated with treatment increase. The total annual cost in the USA is estimated at $17,277 USD for patients with no cirrhosis, $22,752 for patients with compensated cirrhosis and $59,995 for patients with end-stage cirrhosis [7]. The primary clinical manifestations of cirrhosis are impaired function of hepatocytes, the main cell type of the liver, and increased intrahepatic portal hypertension, often resulting in hepatocellular carcinoma [8]. Cirrhosis and its linked vascular dysfunction were long viewed as an irreversible, permanent complication, however advances in medicine suggest that stabilization or even reversal of the cirrhosis may be possible [8, 9].

In humans, the extent of chronic liver disease is categorized into four stages to determine the course of treatment, partly depending on which zone of the liver is damaged [8]. In the first stage, the damage will trigger inflammation of the liver tissue. This is followed by fibrotic deposition and scarring, at which point the liver is in the second stage and normal function begins to decrease. With the continued accumulation of fibrotic tissue the liver enters the third stage; normal function is permanently lost, leading to symptoms such as discoloration of the skin and eyes, loss of appetite, weight loss, and fatigue [10]. Cirrhosis is considered the end (fourth) stage of disease as the liver is unable to produce essential biomolecules like proteins, anticoagulants and detoxify substances, resulting in hypoproteinemia and accumulation of toxic substances in the body [11]. The associated vascular distortion causes the portal and arterial blood supply to expand into the hepatic outflow (main veins), disrupting the connection between hepatic sinusoids and the surrounding liver parenchyma and causing further injury [12]. Current treatment of cirrhosis focuses on symptom management and addressing the underlying causes. In cases of advanced liver disease in which hepatocyte loss cannot be compensated for, hepatocyte transplantation or orthotopic liver transplantation (OLT) are the only clinical options. Hepatocyte transplantation can be used to treat acute liver failure but has a low rate of successful engraftment (less than 30%) [13]. While OLT can restore liver function and prolong the life of a patient, there are drawbacks such as donor shortages, and complications associated with life-long administration of immunosuppressants [14].

## Normal liver physiology and structure

The liver is the largest solid organ in the body and is responsible for many crucial roles associated with metabolism and filtering toxic agents. The basic functional unit of the liver, known as a lobule, consists of a hexagonal space populated by cells of the liver and supplied by the portal triad vessels [15]. Lobules are mainly composed of hepatocytes with distinct membranes which divide the liver into three major zones based on functionality: Zone I, also known as the periportal zone, which supplies oxygenated blood and nutrients to the hepatocytes. This region also plays a crucial role in the formation of bile, cholesterol and proteins. Zone II,the central part of the lobule, consisting of hepatocytes which connect Zone I and Zone II and coordinate lobule function. Lastly Zone III, which contains hepatocytes that participate in lipogenesis, glycolysis and detoxification. This zone is poorly supplied by oxygenated blood due to its location in the distal part of the portal region, rendering it particularly susceptible to ischemia during liver disease [16].

The liver is composed of four main types of cells categorized as either parenchymal, i.e., hepatocytes (HCs), or non-parenchymal**:** hepatic stellate cells, Kupffer cells (KCs), and liver sinusoidal endothelial cells (LSECs), which cooperate in maintaining liver function. Hepatocytes, which constitute 70% of total liver cells, play a major role in detoxification, glycolysis, ketogenesis, lipogenesis, glucose and cytochrome P-450 synthesis, coagulation, and complement factor secretion [17–20]*.* Hepatocytes can also undergo some degree of self-renewal through cell proliferation [21]. Of the non-parenchymal cells, KCs account for almost one third in the liver. Kupffer cells are the resident macrophages of the liver, playing a role in hose immune defense and phagocytosis [22, 23]. Hepatic stellate cells, which compose 5% of liver cells, play a central role in the storing of vitamin A and lipids, but upon injury differentiate into myofibroblasts which contribute to scarring [24]. Lastly, LSECs, which form the largest number of liver non-parenchymal cells (50%), isolate the underlying hepatic stellate cells from the sinusoidal lumen [25]. There are two additional cell types that may play a role in the response to chronic liver disease, although their presence in the human liver is debated. Hepatic stem cells (HSCs), which are a mesenchymal population of liver cells, respond to chronic hepatocellular damage by expressing morphogens such as Wnt and Hedgehog (Hh), and are suggested to support liver regeneration by mobilizing the hepatocellular reserve through the expression of β-catenin and hepatocyte growth factor (HGF) [26, 27]. Lastly, hepatic oval cells, which are a small subpopulation found in the periportal zone (Zone I) in mice [28]. Oval cells are thought to be bipotential, able to differentiate into hepatocytes or bile ductular cells and expressing both biliary and hepatocytic genes. Upon liver injury they are reported to interact with HSCs and secrete acidic and basic fibroblast growth factor (FGF-1 and FGF-2) and vascular endothelial growth factor (VEGF) [3, 29, 30].

## Challenges for liver regeneration

When the liver experiences acute damage, it may experience some degree of regeneration depending on the injury severity. Restoration of the liver following acute damage is mediated by the inflammatory reaction, matrix remodeling (i.e., matrix synthesis and fibrolysis), and hepatocyte proliferation. Non-parenchymal cells are also important for the response to drug toxicity and inflammation. The first phase of liver regeneration is priming, in which hepatocytes are activated in response to growth factors. Work done to characterize liver regeneration in mouse models has demonstrated that shortly after a partial hepatectomy (PH), the expression of over 100 genes in hepatocytes is increased [31]. Following this, there is activation of growth factor receptors expressed by hepatocytes such as epidermal growth factor receptor (EGFR) and c-Met. It has been demonstrated that signaling by these factors is necessary for regeneration; knocking down these genes fully abolishes the regenerative process and results in ascites and hyperammonemia in experimental animal models [32]. Studies have demonstrated that tumor necrosis factor alpha (TNF-α) is also an important factor in promoting liver regeneration following PH [33]. After hepatectomy, TNF-α and other cytokines are upregulated in Kupffer cells [34], along with enteric-derived lipopolysaccharide (LPS) [35]. This activation requires the MyD88 adaptor protein, which is engaged in Toll-like receptor signaling pathways leading to hepatocyte proliferation [36]. Another molecule, lymphotoxin-α (LT-α), supports the activation of TNFR1 on hepatocytes; mouse models lacking TNF-α and LT-α exhibit reduced regeneration capability [37]. Cytokines such as interleukin-6 (IL-6) are also involved in liver regeneration. Elevated IL-6 serum levels are observed early after PH [31] which contribute to the regeneration of hepatocytes, and knocking down IL-6 in the injured liver leads to necrosis and impaired regeneration [38]. Regeneration in IL-6-deficient mice can be rescued by treating animals with stem cell factor (SCF) [39]. Other soluble factors such as FGF and VEGF are crucial signals for angiogenesis, vasculogenesis and lymphogenesis in the liver [40, 41]. Lastly, expression of HGF in the liver has been shown to increase hepatocyte proliferation and accelerate liver regeneration [42]. Other factors that support the regeneration of hepatocytes include: Insulin-like growth factor (IGF), Wnt, Jagged, Delta and Notch, transforming growth factor-β (TGF-β), activins, and bone morphogenetic proteins (BMPs) as discussed in more detail in Friederike Bohm et al. [33].

While many factors are released following liver injury that promote repair and regeneration, there are also many inhibitory factors. In animal models, the ability of the liver to regenerate is compromised in aged individuals (18–24 months compared to 5 to 10-week-old mice). Studies report that this decrease in regenerative ability in the aged liver is due to a reduction in the rate of regeneration with a lack of induction of hepatocyte proliferation factors concurrent with inhibition of cell cycle genes. Following EGF stimulation in aged mice, one group reported a 60% decline in EGF binding to hepatocytes, reduced expression of the hepatic high affinity EGF receptor, and a block between G1 and S-phases of the cell cycle all contributing to a reduction in regenerative capability [43]. Following liver injury there are also certain factors e.g., transforming growth factor-β (TGF-β), interleukin-1 (IL-1), interferon-gamma (IFN-γ), granulocyte colony-stimulating factor (G-CSF) and myeloid differentiation factor 88 (MyD88) involved in the inhibition of hepatocyte proliferation [44]. TGF-β is the most well-characterized inhibitor of hepatocyte proliferation. Early studies show that TGF-β is mainly secreted by non-parenchymal cells during liver regeneration, including hepatic stellate cells, KCs and platelets, to regulate the proliferation of hepatocytes in a paracrine manner [2, 45]. A study by Hanan Saleh et al demonstrated that TGF-β expression induced by EGF inhibits synthesis of DNA in adult rat hepatocytes in primary culture [46]. TGF-β induces apoptosis and may be necessary for apoptosis activation or epithelial-mesenchymal hepatocyte transition in the damaged liver [47]. Recent studies have also shown that inhibiting the TGF-β pathway with the TGF-β type I receptor kinase inhibitor enhances hepatocyte proliferation during acute liver damage [48] and that TGF-β derived from platelets suppresses liver regeneration after PH in rats [49]. Lastly, in a porcine study, the blockade of TGF-β1 with monoclonal antibodies improved liver regeneration [50].

Due to the barriers preventing liver regeneration, alternative therapies are being explored for patients suffering from chronic liver disease and cirrhosis. Regenerative medicine strategies such as cell transplantation are currently among the most promising candidates. Advances in understanding of liver regeneration biology, stem cells, and 3D tissue engineering scaffolds have driven progress toward successful therapies [51]. For patients who have less advanced liver disease, regenerative strategies such as cell transplantation are an attractive alternative treatment that may promote recovery of liver function.

## Cell types for transplantation

The field of cell transplantation has exploded in recent decades as a promising solution to restore function to tissues that are unable to regenerate. Chronic liver cirrhosis is a viable target for transplantation strategies as delivered cells may postpone the need for OLT and reduce the severity of liver damage by both replacing lost hepatocytes and stimulating the remaining hepatocytes to proliferate. The aim of regenerative medicine approaches is to increase the amount of functioning tissue available to restore lost liver function. It can be accomplished by:

1. Promoting the survival and proliferation of existing hepatocytes.
2. Regenerating new tissue to replace damaged cells.
3. Create a growth-permissive environment for the survival and integration of new cells into the host.

A wide variety of cell types can be transplanted to achieve these aims, from primary hepatocytes to stem cells that must be differentiated either prior to transplantation or once in the liver. These cells can be transplanted as a 2D sheet, 3D suspension, or 3D organoid/organized structure [52].

### Hepatocytes

With the aim of restoring function to the liver, primary hepatocytes remain the first choice for liver transplantation. However, prolonged culture of primary human hepatocytes is unreliable and results in a loss of morphology and liver-specific function. Porcine hepatocytes, while easier to obtain, have restricted biocompatibility and may elicit an immune response. Lastly, human fetal hepatocytes, while able to withstand longer culture periods, are also difficult to obtain and carry tumourigenic potential [53]. Ongoing research is investigating methods to maintain hepatocyte phenotype in culture, such as culture on a monolayer coating of collagen, which has been demonstrated to rescue hepatocyte phenotype [54], but without similar strategies for the transplantation of hepatocytes, researchers must turn to other sources of cells.

### Hepatic stem/progenitor cells

Hepatic stem/progenitor cells are self-renewing bipotent cells endogenous to the liver. They are derived from Lgr5^+^ (mouse) or EpCAM^+^ (human) cholangiocytes and from bile duct fragments from mouse and human livers with the ability to differentiate into hepatocytes or cholangiocytes [55, 56]. Another type of hepatic stem/progenitor cell are oval cells, identified in rodent studies and named for their oval-shaped nuclei [57]. Oval cells can be detected after induction of liver damage in models of toxin-induced injury or hepatic carcinogenesis. The source of oval cells in the liver is unknown, but is hypothesized to be billary epithelial cells [31]. While the lineage of these cells is well suited for liver transplantation, they are a rare cell type and there is no standardized protocol for their in vitro maintenance and differentiation into mature hepatocytes [58]. There are also no means of direct comparison of maturation level or phenotypic characterization of the derived hepatocytes, making it difficult to compare results between studies. During development, hepatoblast differentiation into hepatocytes is regulated by numerous chemical and biophysical factors [59]. These cues may be studied further and used as a basis to develop differentiation protocols in vitro, which is currently under study [58, 60].

### Non-hepatic stem cells

The field of cell transplantation has employed a variety of stem cell types to restore function in many organ systems and the liver is no exception to this. Embryonic stem cells (ESCs), mesenchymal stem cells (MSCs) and human induced pluripotent stem cells (hiPSCs) are all capable of differentiating into functional hepatocyte-like cells when the appropriate biophysical and chemical cues are provided and may yield an unlimited supply of hepatocytes [61–63]. However, it is difficult to fully recapitulate all characteristics of mature hepatocytes with these sources of hepatocyte-like cells [64]. ESCs, while possessing an impressive capacity for proliferation, do not offer the same potential of personalized medicine that can be achieved with MSCs or iPSCs harvested from the patient’s own body for transplantation. MSCs can be harvested from the bone marrow, adipose tissue, and umbilical cord blood and have been shown to differentiate into hepatocyte-like cells in vitro and in vivo [65]. A recent meta-analysis conducted on 14 human umbilical cord blood MSC transplantation trials (a total of 717 liver cirrhosis patients) reported that MSC transplantation was correlated with improved liver function and clinical symptoms with no severe events associated with transplantation [66]. There is a need for a reliable source of hepatocyte-like cells, which may be met with the use of hiPSCs due to their advantages over ESCs for in vitro hepatocyte differentiation and maturation [67, 68]. Hepatic progenitor cells or hepatocyte-like cells can be derived from hiPSCs or transformed directly into hepatocytes from fibroblasts [69] and show liver-specific gene expression and function both in culture and following transplantation in animal models of liver disease [70, 71]. The development of in vitro protocols result in functional, mature hepatocyte-like cells for transplantation is currently underway. It is important to note however that the somatic primary source of iPSCs can determine their ability to be directed towards a hepatocyte phenotype [72–74]. Interestingly, a recent publication reported the development of a non-human primate model of severe liver fibrosis that faithfully replicates human pathophysiology [75]. The authors transplanted hiPSC-derived hepatocyte-like cells via the portal vein using a clinically-relevant protocol and evaluated outcomes after 14 days. Although only assessed short-term, they reported albumin-positive viable cells in the interlobular connective tissue around the portal area vessels. This study lays the foundation for further use of non-human primates to investigate the feasibility of cell transplantation strategies that are currently limited to rodent studies.

### Multiple cell types

Given that no one cell type can meet all the requirements for successful regeneration, many researchers have opted to simultaneously transplant multiple cell types. This strategy can involve the transplantation of one type that will provide liver functioning and one or more secondary types to provide paracrine support. For example, the addition of endothelial cells can improve hepatocyte function and facilitate vascularization after transplantation, as was demonstrated by Du et al., [62]. This study encapsulated iPSC-derived endothelial cells (ECs) and hepatocytes into hydrogel fibers to mimic the in vivo spatial configuration of the liver [62]. A similar study combined primary hepatocytes and endothelial cells and found that hepatocytes exhibited higher bioactivity and liver-specific function when combined with ECs compared to culturing alone [76]. One study combined three cell types – hiPSC-derived hepatic progenitor cells, HUVECs (human umbilical vein endothelial cells), and MSCs – into a 3D printed scaffold with microscale hexagonal architecture to mimic a liver lobule. Although the authors found that the combination of three cell types enhanced liver-specific gene expression and hepatocyte function after 10 days in culture, they did not test their scaffold in vivo [77]. Future multi-cell type strategies such as this will need to expand their applicability for in vivo use.

## Biomaterials for cell transplantation to the liver

**Fig. 1:**
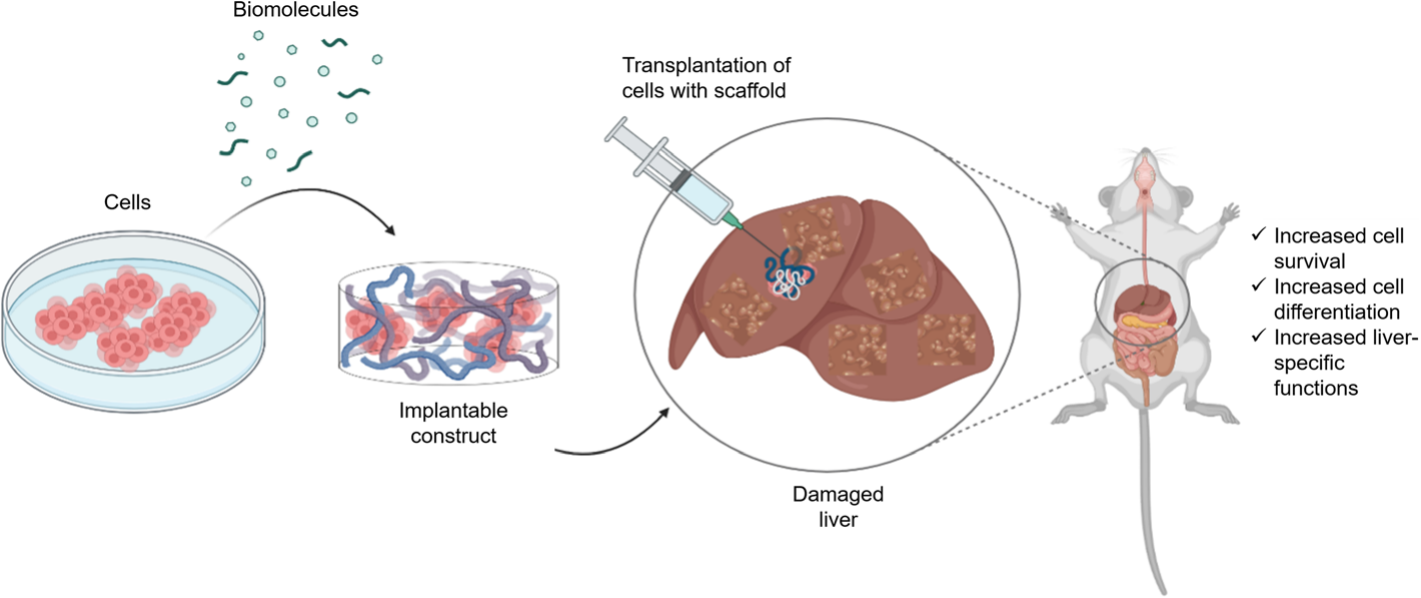
Biomaterials can be combined with cells and biomolecules for transplantation to treat chronic liver disease. Biomaterials are injectable, biocompatible, degradable and provide physical support and biological cues to transplanted cells to promote their survival and differentiation. Created with BioRender (www.biorender.com)

Current cell transplantation strategies are hampered by poor post-delivery survival, a loss of supporting ECM and vasculature in liver, and difficulty in achieving maturity and incorporation into the host tissue [78]. Due to these hurdles, researchers have turned to the delivery of cells within biomaterial constructs to support transplanted cells and provide a scaffold for regeneration following transplantation (Fig. 1). Evolving scaffold manufacturing techniques has led to the development of matrices to promote liver regeneration from basic porous scaffolds to more complex organized scaffolds [79] (Table 1). Biomaterials need to at least partially re-create the main features of endogenous ECM 3D microarchitecture, stiffness, protein composition, and proangiogenic properties. Aggregation of hepatocytes is reported to be important to increase cell viability, function and long-term phenotypic stability [111]. This can be facilitated with the use of a biomaterial that can support hepatocyte aggregates in culture which can then be transplanted in vivo. The use of biomaterials also allows for the implantation of cells directly into the liver rather than through the portal vein, ensuring a higher number of cells are delivered to the site of liver regeneration [52].

**Table 1:** Summary of natural and synthetic materials used to culture and deliver cells for liver

Material	Preparation	Cell Type	Effects	Ref
***Natural Materials***				
Alginate	Freeze drying + calcium crosslinking	Rat hepatocytes	**In vitro**: increased cell viability; hepatocyte spheroid formation; increased urea synthesis	[80]
	Freeze drying + calcium crosslinking	Human hepatocytes and non-parenchymal cells	**In vitro**: expression of mature liver enzymes; albumin secretion; liver organoid formation by 6 wks capable of drug metabolism	[81]
	Freeze drying + calcium crosslinking	Porcine bone marrow-derived MSCs	**In vitro:** expression of liver-specific genes and proteins; albumin and urea production; 56.7% of cells expressed cytokeratin-18	[82]
	Gelation freeze technique	Rat hepatocytes	**In vitro:** cell viability maintained; albumin and urea production; fibronectin synthesis; no cell proliferation	[83]
	3D bioprinting + calcium crosslinking	HepG2 cell line	**In vitro:** liver-specific gene expression; recapitulation of lobule structure	[84]
	3D bioprinting + calcium crosslinking	Mouse embryonic fibroblasts	**In vitro:** formed hepatocyte-like colonies; **In vivo:** transplantation in damaged liver expressed liver-specific markers, survived for up to 28 d	[85]
Hyaluronate	Photo-crosslinking	Fetal liver cells	**In vivo**: regeneration of tissue; prevention of jaundice and production of albumin; moderated liver fibrosis	[86]
	Esterification + hydrolyzation	Mouse hepatocytes	**In vitro:** established cell-cell contacts and albumin secretion in culture after 14 d; **In vivo**: survival of transplant for 35 d	[87]
Chitosan	Freeze drying + fructose addition	Mouse hepatocytes	**In vitro**: formation of cellular aggregates; albumin and urea secretion	[88]
	Electrospinning	Human hepatocytes	**In vitro:** formation of aggregates, liver-specific function maintained; easy detachment for downstream applications	[89]
Collagen	Coated onto dextran microcarriers	Rat hepatocytes	**In vivo:** survival and liver-specific function in rats lacking bilirubin metabolism	[90]
	Coated onto synthetic membrane	Porcine hepatocytes	**In vitro:** cell proliferation and synthesis of albumin and urea	[91]
	3D bioprinting	HUH7 cell line	**In vitro:** interconnected scaffold 3D geometry increased cell viability and proliferation; increased liver-specific function	[92]
Gelatin	3D bioprinting	Rat hepatocytes	**In vitro:** viability and liver-specific functions maintained for two months	[93]
Chitosan-gelatin	Freeze drying	HepaRG, LSEC, and HUVEC	**In vitro:** HepaRG cells best viability and liver-specific function when cultured with LSECs; 3D culture improved results vs 2D	[94]
	Freeze drying	Mouse hepatocytes	**In vitro:** combination with alginate best albumin secretion; formation of spheroids; decrease in cell viability by 10 d	[95]
Cellulose	Phase separation and lyophilization	Rat hepatocyte	**In vitro:** formation of hepatocyte spheroids; liver-specific functions; mature hepatocyte phenotype	[96]
	Infused into PLLA scaffold	Human iPSC-derived hepatocytes	**In vitro**: liver-specific cellular function	[97]
Heparin	Photopolymerization + lithography	Human ADSCs	**In vitro:** increased albumin and glycogen storage; **In vivo:** liver retention and functional recovery	[98]
	Lipid conjugated + coated onto cells	Human ADSCs	**In vivo:** lowered AST/ALT levels, increased hHGF, reduced inflammation, cell retention	[99]
Natural ECM	Decellularization +3D bioprinting	HepG2, BMMSCs	**In vitro**: induced stem cell differentiation; enhanced HepG2 cell function	[100]
	Decellularization + Ag nanoparticles	HepG2 and EAhy926 cell lines	**In vivo**: proliferation and HGF expression; lower aspartate transaminase and alanine transaminase plasma levels; lower liver homogenate nitric oxide levels	[101]
	Gelation of liver-derived ECM powder	Primary human hepatocyte	**In vitro**: high levels of albumin expression and secretion, ammonia metabolism, and hepatic transporter expression and function	[102]
	Decellularization (compared 4 methods)	Human hepatic stem cells	**In vitro**: lost stem cells markers; differentiated and maintained parenchymal phenotype for 8 wks	[103]
***Synthetic materials***				
PLLA	Dissolved in organic solvent	Rat hepatocytes	**In vitro:** formed spheroids with intercellular junctions; hepatocyte morphology and function preserved	[104]
	Particulate leaching method	Rat hepatocytes	**In vivo**: 1 wk. post-implantation: improved cell survival, glycogen storage capacity maintained	[105]
	3D printing + infused collagen	Human iPSC-derived hepatocytes	**In vitro**: viability; polarization; formation of bile canaliculi-like structures; natural ECM superior hepatocyte-specific function compared to PLLA scaffold	[97]
	Gas foaming	Fetal liver cells, hepatic parenchymal cells	**In vitro:** maintained cell viability; stimulated maturation of hepatic parenchymal cells	[106]
PCL	Electrospinning	HepG2 cell line	**In vitro**: cells produced ECM; In vivo: support hepatic phenotype and function	[107]
	3D printing + collagen in channels	Hepatocytes, HUVECs, human lung fibroblasts	**In vitro:** improved survival of hepatocytes; albumin and urea secretion; formation of network with non-parenchymal cells	[108]
PCL-PLGA	Multihead 3D printing	Rat hepatocytes	**In vitro:** improved cell adhesion and proliferation; high viability	[109]
PEG	Photopatterning	Hepatocytes, Lewis rats, NIH 3 T3-J2	**In vitro:** improved viability and liver-specific function over unpatterned controls	[110]

### Biomaterial properties

There are many biophysical factors which are important in selecting a material for cell transplantation. We have chosen here to highlight three of particular relevance for liver transplantation.

#### Porosity

Both the size and geometry of biomaterial pores are important for cell delivery, with porosity affecting cell adhesion, proliferation, migration and differentiation. Biomaterial porosity and pore size can be tuned by adjusting the concentration or crosslinking density, and reports in the literature generally suggest a pore size of 50 – 200 μm for the culture of hepatocytes [52, 94]. Interconnected pores of approximately 50 – 70 μm in an alginate/chitosan 3D scaffold resulted in higher hepatocyte-specific function than the control [112] and highly porous alginate/chitosan hydrogels with pore sizes of 100-200 μm support the growth of hepatocyte spheroids in culture [113, 114]. One study systematically tested the response of rat primary hepatocytes to pore sizes of 10, 18, and 82 μm using collagen foam scaffolds, finding that subcellular pore diameters resulted in cells taking on a cuboidal morphology and forming a monolayer, whereas increasing the pore size to 82 μm induced cell spreading into a 3D network and increased albumin secretion [115]. In addition to hepatocyte function and viability, pore properties can influence the behaviour of other cell types. For example, angiogenesis can be influenced by pore size and porosity [116], as well as hepatic stellate cell migration on PLGA scaffolds [117].

#### Stiffness

Biomaterial stiffness is important for both the cells which are seeded as well as the location of transplantation. Stiffness has a direct effect on cell adhesion to the scaffold, cell aggregation, cell motility, and growth factor responsiveness, as well as parenchymal and nonparenchymal interactions [118, 119]. In the liver, stiffness increases as liver disease progresses and the tissue becomes more fibrotic; the stiffness of normal, early, and late stage fibrosis liver is 1.5–4.5 kPa, 4.1–12.9 kPa, and 16.3–48 kPa, respectively [120]. Biomaterials can be tuned to match the stiffness of the healthy liver, or to the stiffness of various pathological states for in vitro study [121]. In general, hepatocyte function decreases as stiffness is increased [122], although there are some inconsistencies reported in the literature which may be due to variability in material preparation and properties. For example, increasing the stiffness of a polyelectrolyte multilayer decreased albumin production of hepatocytes, but in polyacrylamide gels, increased stiffness led to better hepatocyte function [123]. The hepatocellular responses to stiffness include cell migration, proliferation and differentiation. Culture of primary human hepatocytes evaluated at stiffnesses ranging from 600 to 4600 Pa using crosslinked HA scaffolds with liver ECM showed better attachment, viability and organization of actin cytoskeleton with increasing stiffness, however superior hepatocyte function was reported at 1200 Pa [124]. This difference could be due to the differing molecular pathways that control cellular functions and their responses to changes in stiffness. For example, hepatocyte function was tested with three stiffnesses representing normal (4.5 kPa), early fibrosis (19 kPa) and late fibrosis (37 kPa) using a polyvinyl alcohol hydrogel. The researchers reported a dynamic balance of β-1 integrin and β-catenin pathway activation/deactivation that is controlled by stiffness, which modulates the cytoskeleton and thus migration of hepatocytes. Stiffness of a biomaterial can also control cellular differentiation. This was investigated using methacrylated HA hydrogels coupled with RGD peptides which are able to form a soft gel via Michael-type addition but could be stiffened by light-mediated radical crosslinking [125]. When hepatic stellate cells were cultured in this hydrogel, they reversibly assumed a myofibroblast phenotype with in situ stiffening. Inducible crosslinking biocompatible materials like this and others can be used to study the dynamic phenotype changes in response to fibrosis in liver disease. They may also be applicable to in vivo transplantation wherein low viscosity hydrogels can be injected and then crosslinked in situ to increase the stiffness to that of the normal liver in a minimally-invasive manner.

#### Geometry

Biomaterials can be fabricated using many different methods, such as electrospinning into nanofibers, 3D printing, freeze drying to form a scaffold, hydrogel, or microsphere formation. The resulting 3D geometry of the material will greatly influence the viability, proliferation, and function of seeded cells. For example, the thickness of nanofibers – which can be made from synthetic, natural, or self-assembling peptides - will control cell adherence and survival [126]. Nanofibers and microspheres provide increased surface area for cell adhesion [127] and can promote 3D hepatic sheet formation [128]. Hepatic lobules are roughly hexagonal in geometry, and micropatterning techniques to fabricate scaffolds that mimic the in vivo structure of hepatic lobules can be used to culture and maintain hepatocytes with other cell types prior to transplantation [129, 130]. Pore geometry is also important, as illustrated in a study by Lewis et al., [92] who tested varying scaffold pore geometries while keep pore size consistent using 3D printed gelatin scaffolds seeded with an undifferentiated hepatocyte cell line. Interestingly, they found that cell survival and proliferation did not differ between geometries, but that a more interconnected pore geometry allowed hepatocytes to aggregate, increasing albumin and cytochrome P450 production in vitro. Lastly, an important feature of hepatocytes is the polarization of cells which maintains junctional components and apical transporters necessary for cargo transport [131]. Polarization can be achieved using a sandwich system wherein cells are cultured on a 2D hydrogel layer with a second layer placed on top, which results in improved hepatocyte function in vitro [131, 132].

### Natural materials

#### Hyaluronic Acid (HA)

Hyaluronic acid (HA) is a widely used natural material for cell transplantation due to its biocompatible and tunable nature. It is abundant in environments of increased cell proliferation such as embryogenesis, wound repair, and regeneration. For the liver, HA is the main component of the perisinusoidal space and combined with its high hydrophilicity to form a hydrogel, this makes HA a highly biocompatible material for liver transplantation [133]. High molecular weight HA also exhibits anti-inflammatory properties in many models, a feature which may reduce the inflammation associated with liver disease and make the liver environment more hospitable to transplanted cells [134]. Furthermore, the backbone of HA contains carboxyl and hydroxyl groups that can be functionalized with other molecules for crosslinking to tune hydrogel stiffness, or the addition of pro-survival factors [135]. Numerous groups have demonstrated the efficacy of HA for the delivery of different cell types to the liver. Turner et al.*,* grafted human hepatic stem cells in thiol-modified carboxymethyl HA scaffolds and transplanted them into a mouse model of liver injury [136]. The use of modified HA allows for the regulation of crosslinking and thus stiffness through the addition of a PEGDA (Poly (ethylene glycol) diacrylate) crosslinker. The researchers combined HA with either type III collagen or laminin and demonstrated that compared to direct or vascular injection of cells without a scaffold, the use of the HA scaffold resulted in a greater number of engrafted cells that remained localized to the liver and survived for 3 months post-transplantation [136]. A HA-based scaffold can also support the post-transplantation viability of multiple cell types and the formation of de novo vascular networks, as demonstrated when scaffolds seeded with hMSCs and rat hepatocytes were preconditioned in a bioreactor system prior to implantation [137]. Aside from hydrogels, HA has also been tested in the form of a sponge; Katsuda et al., (2010) transplanted fetal liver cells encapsulated in a HA sponge into mesenteric blood vessels of a rat model of Wilson’s disease. They reported that, in addition to successful engraftment, the transplantation site of the mesentery provided a ready blood supply that supported transplanted cells, increasing albumin production, preventing jaundice, and diminishing fibrosis in treated animals [86]. One caveat that may need to be considered for future use of HA is that low molecular weight HA production is upregulated during liver fibrosis in human and mouse, promoting a pro-fibrogenic phenotype, proliferation, and invasion of hepatic stellate cells via TLR4 and CD44 signaling [138]. This suggests that although there are many benefits to the use of HA, further work is needed to characterize the host response to HA.

#### Chitosan

A unique bio-based polysaccharide, chitosan is obtained from N-deacetylation of chitin. It possesses antibacterial activity, mucoadhesive and analgesic properties [139], and is degraded into non-toxic byproducts which can be tuned by adjusting properties such as molecular mass and extent of deacetylation [140–142]. Due to the lack of cell binding domains and thus weak bioactivity towards cells, as a material for cell transplantation chitosan is often combined with other materials or functional molecules [52]. For example, lactose moieties can be conjugated to chitosan to promote cell attachment and tune the material mechanical properties, resulting in improved cell adhesion, biocompatibility and mechanical stability in a 3D scaffold for hepatocytes in culture [143]. Surface-galactose ligands can also be used to modify a chitosan nanofibrous scaffold. This modification slowed degradation and conferred biocompatible mechanical properties onto the material, leading to enhanced bioactivity of primary hepatocytes in vitro [89].

#### Alginate

Like chitosan, alginate is also a naturally-derived biocompatible polysaccharide not found in mammals. It is abundant and inexpensive, but due to its low cell-adhesiveness is often combined with other materials to increase tunability of its properties and support of transplanted cells [144]. For example, it can be both crosslinked to form hydrogels containing disulfide bonds which allows for cleavage under physiological reducing conditions, and combined with chitosan via surface modification for improved mechanical properties [145]. An RGD-modified-chitosan-alginate polyelectrolyte complex fibrous scaffold was demonstrated to support the delivery and survival of human MSCs in a hepatectomy rat model. Cells were first transdifferentiated into hepatocyte-like cells in vitro prior to seeding on the scaffold and were then implanted onto the liver. The researchers reported that transplantation in this scaffold resulted in increased cell survival, production of human albumin, and maintenance of the differentiated hepatic phenotype after 14 days [146]. Alginate has also been used to fabricate microbeads to encapsulate hepatocytes for in vivo implantation. One group demonstrated that microbeads of approximately 600 μm in size were able to support viability of human hepatocytes following intraperitoneal transplantation into a rat model of acute liver failure [147]. They demonstrated safety of the technique, mitigation of the severity of liver damage, and no significant immune response after 7 days compared to empty microbeads. One question not addressed by this work is whether microbeads would degrade over time, allowing migration of hepatocytes out into the tissue where they may repopulate the liver, or whether this strategy relies more on secretion of factors that promote recovery by the sequestered hepatocytes. It has been previously reported that within microcapsules, the mobility of hepatocytes is dependent on the viscosity, which will determine if hepatocytes aggregate within the capsule or are able to migrate outward [148]. In order to mimic the liver microenvironment using a 3D culture system, another group fabricated galactosylated alginate-based microcapsules for hepatocyte culture. Galactose groups are reported to improve the function of hepatocytes as they recognize galactose as a ligand. The researchers demonstrated that albumin secretion and urea synthesis of primary human hepatocytes was maintained in the microcapsule system [113].

#### Gelatin

Collagen, while the most abundant component of the ECM, must be used at high concentrations for sufficient stiffness to support cells, making it cost ineffective, and degradation is faster than polysaccharides and synthetic polymers, so has limited application for liver transplantation although it has been used for hepatocyte culture successfully [52, 149, 150]. However, gelatin, which is a natural component of the ECM derived from denatured type I collagen lacking the three alpha-helix structure, is often used in place of collagen [151]. Gelatin maintains the composition of collagen, is biodegradable, inexpensive and easy to fabricate into a 3D scaffold by various methods alone or in combination with other biomaterials. To date, gelatin has mainly been used as a 3D porous scaffold to support in vitro hepatocyte culture [152, 153]. Hou and Hsu fabricated a composite glutaraldehyde chitosan/gelatin 3D scaffold by freeze-drying a solution of the biomaterials. They were able to tune the compressive modulus and obtained pore structures similar to the liver ECM and found that their scaffold supported hepatocyte viability and function in culture. They reported that after 1 week, cells expressed higher levels of albumin and urea when the pore size was 150-200 μm [154]. Others have combined gelatin with galactose or laminin to support the differentiation and viability of hepatocytes in culture, finding that 3D gelatin generally increases the expression of hepatocyte-specific genes and markers, as well as functional outputs such as albumin and urea levels [155, 156]. Lastly gelatin can be combined with methacrylic anhydride to synthesize gelatin methacryloyl (GelMA). GelMA is biocompatible, degradable by MMPs, and possesses tunable mechanical properties [157]. It has been used to create 3D lobule-like tissues with hepatocytes and fibroblasts [158], as well as a 3D printed bioink for adult hepatocyte culture [159]. The ability of gelatin and its composites to support transplantation of cells into the liver and maintain the hepatocyte-specific function remains to be demonstrated.

#### Cellulose

Cellulose is a natural material derived from plant cell walls, abundant in nature and easily produced, and thus cost-efficient for fabrication. Cells can adhere to cellulose via hydrophilic hydroxyl moieties and specialized cellulose binding domains [160]. Cellulose is bioactive and has biomechanical properties that are compatible for liver transplantation, although it is infrequently reported in the literature for this purpose [161–163]. Cellulose has, however, been used extensively for hepatocyte culture [164–166]. Madhushree et al.*,* tested the ability of a nanofibrillar cellulose hydrogel to induce spheroid formation and differentiation of hepatic cell lines HepaRG and HepG2 [164]. They reported that without the addition of any other bioactive components the cellulose hydrogel formed a 3D scaffold in situ after injection and promoted hepatocyte spheroid formation and differentiation. One drawback of cellulose is that it cannot be degraded naturally in humans. Some bulk washout may occur, but overall a cellulose scaffold can be considered a permanent construct that cannot be replaced with regenerated native tissue. Conversely, this will provide long-term structural support that could be beneficial if combined with the delivery of cells that can perform the function of hepatocytes. There is also some evidence that cellulose may cause structural damage in the liver and steatosis (fatty liver disease) which should be carefully considered when adapting this biomaterial for cell transplantation to the liver [167, 168].

#### Fibrin

Fibrin and its precursor fibrinogen have been used for liver cell culture and transplantation, albeit to a lesser extent than other natural materials. It has some advantages such as the potential to be harvested from the patient’s blood, reducing immunogenicity [169], but when unmodified it has poor mechanical properties and rapid degradation in the body [170]. Fibrin does however play a role in coordinating hepatocyte division during liver regeneration via crosstalk through αVβ3 integrin, and has been shown to contribute directly to liver regeneration by driving platelet accumulation and regeneration after partial hepatectomy, making it an attractive material to combine with cell delivery [171, 172]. Banihashemi et al.*,* produced fibrin scaffolds that support human hepatocyte growth using fibrinogen and thrombin from plasma. Their scaffolds demonstrated good stability, allowing the culture of human hepatocytes and the formation of a liver-like tissue in vitro [173]. Fibrin has also been combined with HA to form semi-interpenetrating polymer networks to differentiate MSCs in vitro with positive results, but this combination has yet to be tested for the application of cell transplantation for the liver [174]. Future work may include the testing of fibrin as a composite scaffold with other materials to take advantage of its bioactive properties while offsetting its suboptimal mechanical properties.

#### Heparin

Heparin is an anticoagulant glycosaminoglycan naturally found in the blood. It contains moieties that bind to a large variety of growth factors, extending their half-lives and increasing bioactivity [98]. It is known to improve liver regeneration through mechanisms such as accelerated hepatocyte proliferation and can regulate cell growth and differentiation via the concentration of growth factors [175]. Previous work with heparin for liver regeneration has demonstrated its use for encapsulation of cells in a hydrogel scaffold, and to coat cells themselves to prolong their retention and reduce the immune response in vivo [98, 175, 176]. In a series of studies, Hwang et al. first used heparin to form micropatterned hydrogels for the in vitro differentiation of hASCs into hepatocytes and to prepare ‘micropatches’ that could be loaded with hASCs and injected intravenously to promote functional recovery in the liver [98]. The same authors also used lipid-conjugated heparin to coat hASCs prior to transplantation in a mouse model of acute liver failure, reporting that heparin coating increased the serum level of hepatic growth factor [99].

#### Composite scaffolds

**Fig. 2:**
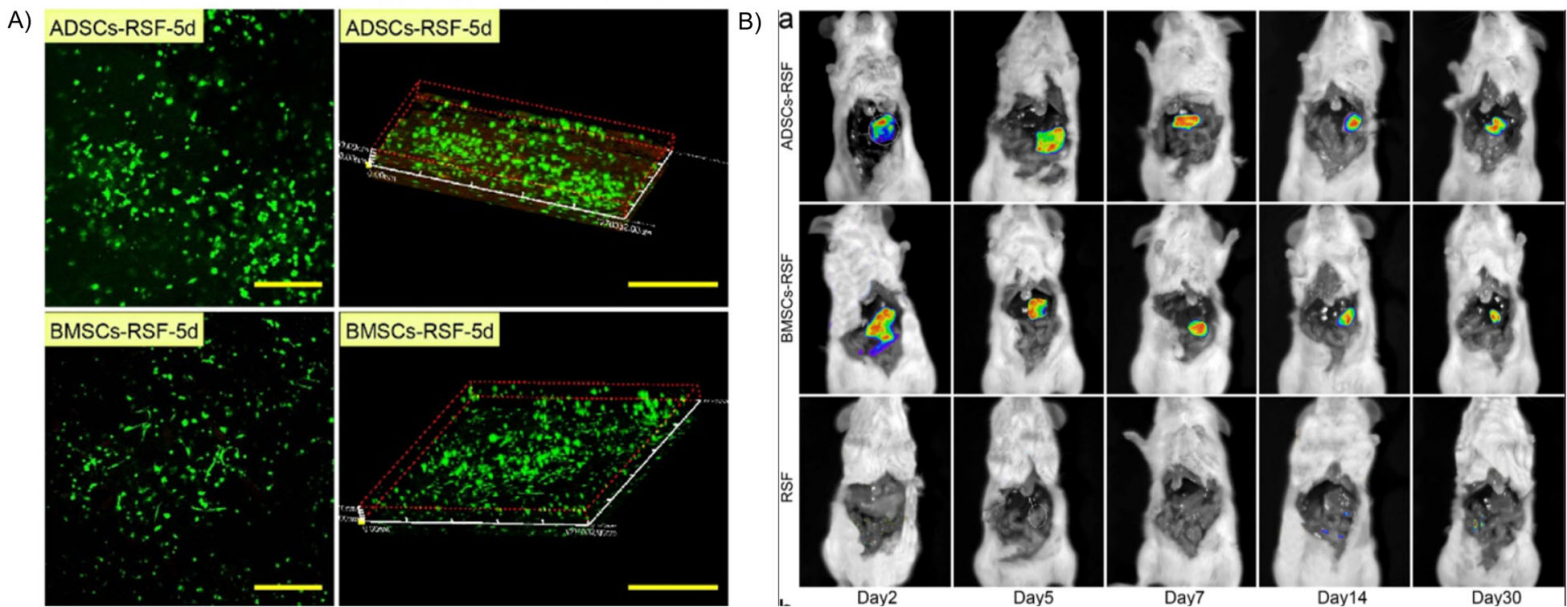
Silk fibroin scaffolds provide support for adipose-derived stem cell (ADSC) and bone marrow stem cell (BMSC) survival and liver-specific function both in vitro and in vivo. **a** 2D and 3D light scattering confocal microscopy images of ADSCs and BMSCs (GFP, green) 5 days after encapsulation in the scaffold. Scale bar = 200 μm. **b** Fluorescence of ADSCs and BMSCs in the silk fibroin scaffolds implanted into mouse livers demonstrating cell survival up to 30 days. Adapted with permission from Ref [182]

A single material cannot meet all the requirements of liver regeneration so many strategies involve a combination of different materials that together can support cell transplantation and regeneration. Many scaffolds blend two different polysaccharides, or a polysaccharide with proteins. As mentioned previously, materials with low cell adhesion such as chitosan or alginate may be combined with other biomaterials such as HA and functionalized to increase cellular interactions [112, 145]. One example of this are porous hybrid sponges formed from galactosylated chitosan and HA for the coculture of primary hepatocytes and endothelial cells [76]. One group studied the combination of galactosylated alginate or unaltered alginate with chitosan and/or collagen I, reporting that the combination of galactosylated alginate + chitosan + collagen I induced the best liver-specific function of HepG2 cells after 10 days of culture [177]. Natural components of the liver ECM can be combined with biocompatible materials from other areas of the body; combining HA and collagen I with hydroxyapatite into a porous scaffold yields higher viability and liver-specific gene expression of primary hepatocytes when cultured long-term [178]. In addition, Matrigel, a composite material derived from ECM of mouse sarcoma tumors, has been used to both culture adult hepatocytes [179, 180] but is hampered by concerns of batch-to-batch variability and undefined composition. Future studies may explore the combination of liver ECM components with other less conventional materials such as xyloglucan, a hemicellulose found in the primary cell wall of vascular plants, which was shown to enhance liver function of a human hepatocyte carcinoma cell line by promoting cell-matrix interactions in an in vitro 3D scaffold [181]. Another potential material is silk fibroin, obtained from *Bombyx mori* cocoons, which has been used extensively for regenerative applications in the nervous system, blood vessels, cartilage, and the bladder. Xu et al., used silk fibroin to deliver MSCs onto the surface of mouse livers with acute failure, where the cell-material combination stimulated neovascularization and improved liver function of animals [182] (Fig. 2).

### Decellularized materials

**Fig. 3:**
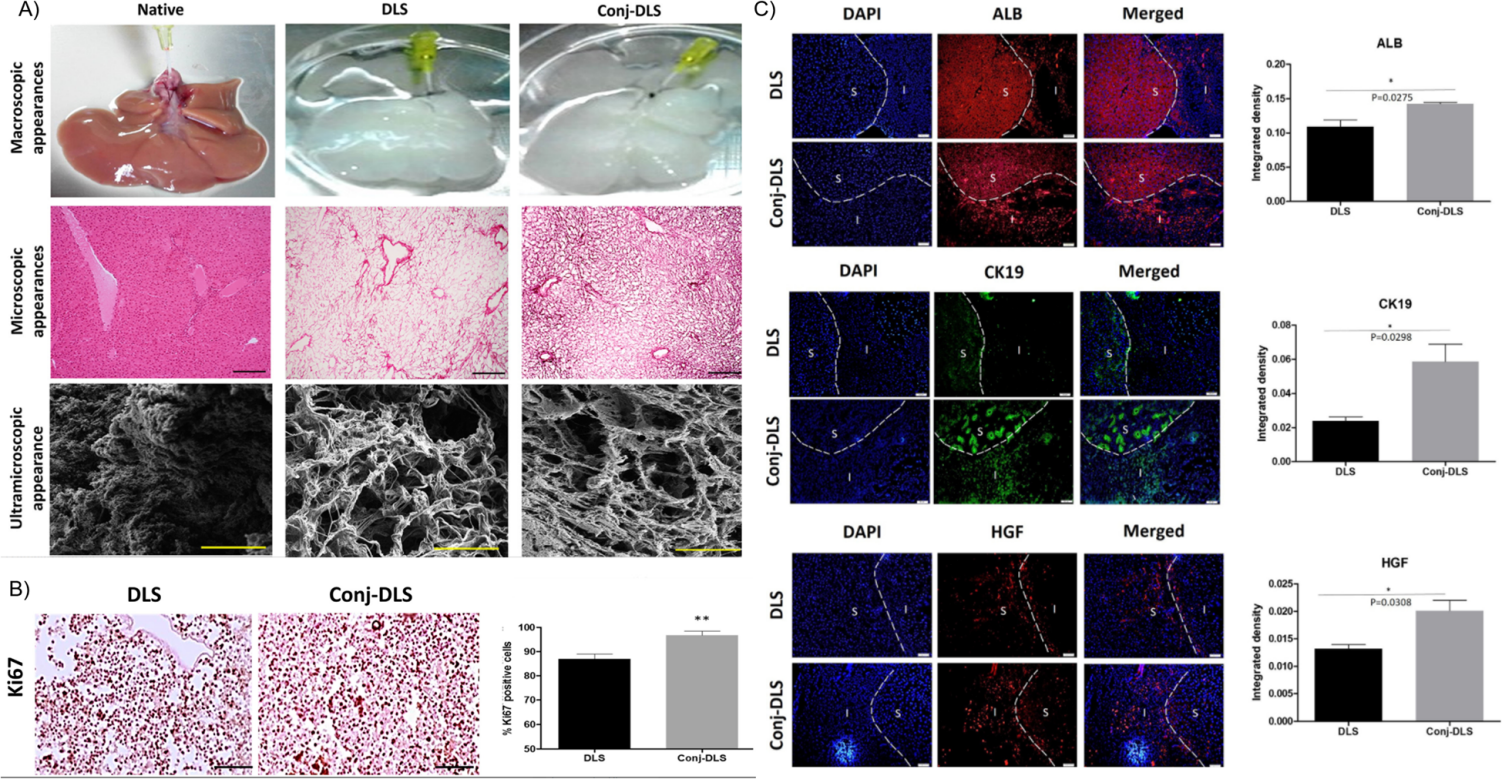
Recellularized liver ECM scaffolds conjugated with homogenized liver ECM promote HepG2 cell proliferation and liver-specific function following transplantation into the mouse liver. **a** Whole liver, H&E, and ultramicroscopic images of decellularized liver scaffold (DLS) with and without conjugated liver ECM (Conj-DLS). Conj-DLS had more ECM and thicker collagen fibres compared to the DLS. **b** Semi-quantitative analysis of Ki67+ cells in DLS and conj-DLS demonstrating higher number of Ki67+ cells in conj-DLS compared to DLS. **c** Representative immunofluorescent images and analysis of ALB, CK19, and HGF expression after hepatic transplantation. I = implanted tissue, s = surrounding liver. Scale bar = 100 μm. Adapted with permission from Ref [189]

The use of decellularized materials for liver regeneration is becoming increasingly recognized as a major strategy for cell delivery. This method involves the isolation of the ECM from tissues by removing cells and leaving the ECM intact, through protocols such as incubation in detergents, freeze/thaw cycles, and perfusion (reviewed in Gilbert et al. [183]. The remaining structure has a chemical composition and architecture that is similar to the native liver, retaining ECM signaling molecules and reducing the immune response against the transplant, and can be reseeded with the desired cell type for transplantation. Whole rat livers can be decellularized and reseeded with hepatocytes and endothelial progenitor cells, as demonstrated by Zhou et al., who injected these scaffolds directly into the parenchyma via portal vein fusion, where the intact vascular structure of the artificial organ is directly connected to the circulation of the recipient [184]. Decellularized rat liver scaffolds can also promote hepatic differentiation of murine MSCs by enhancing the expression of hepatocyte-specific genes and proteins [65]. Alternatively, extracts from acellular liver ECM, or whole fresh liver can be combined with natural polymers such as HA or type I collagen to form a hybrid material [185]. Skardal et al. demonstrated that customized HA hydrogels with liver-specific ECM components may be an efficient method for in vitro expansion of human hepatocytes for cell therapy or drug and toxicology screening purposes [186]. Another group adopted a similar approach by fabricating a liver ECM scaffold containing immobilized HGF and demonstrated that this scaffold could support liver-specific function of hepatocytes in culture [187]. Heparin can also be immobilized onto liver ECM scaffolds by means of layer-by-layer deposition, allowing the spatial organization of factors that can then be combined with primary rat hepatocytes that maintain albumin and urea production in vitro [188]. Future work on decellularized liver scaffolds will need to focus on improving preservation of liver ultrastructure and cell attachment. For example, a promising recent study combined homogenized liver ECM with decellularized liver scaffolds through chemical conjugation, finding that this approach improved cellular spreading, viability, and angiogenesis when the scaffold was transplanted into rat livers [189] (Fig. 3).

### Synthetic materials

Synthetic materials have also been used extensively for cell transplantation to the liver. Unlike natural materials which often have poor mechanical properties, uncontrolled degradation and limited sources, synthetic polymers are readily available, have better mechanical properties, and tunable degradability. The main disadvantages of synthetic materials are the low binding affinity to cells, which can be overcome with the addition of biomolecules, and their degradation products, which may not be biocompatible [149]. Although there are multiple types of synthetic materials, we will limit our discussion here to the use of aliphatic polyesters for liver transplantation. Aliphatic polyesters, polylactic acid (PLA), polyglycolic acid (PGA), poly(Ɛ-caprolactone) (PCL), and their copolymers can be formed by polycondensation, ring opening polymerization, and enzymatic polymerization [190]. The most commonly used monomers for their synthesis in biomedical applications are lactide, glycolide and caprolactone [191]. Aliphatic polyesters contain ester bonds susceptible to hydrolysis, allowing for tunable degradability. However, many degradation products are acidic which can cause an unfavorable microenvironment for cell transplantation [192]. Despite this, these polymers have been widely used for regeneration applications in tissues such as skin, nerve, blood vessel, lung, and liver [193].

#### Polylactic Acid (PLA) and Poly-L-lactic Acid (PLLA)

Polylactic acid (PLA) and its derivative poly-L-lactic acid (PLLA) are copolymers that are biodegradeable and tunable over a wide range of mechanical properties. When degraded, PLA and PLLA form lactic acid monomers that are further metabolized into carbon dioxide and water. They can be formulated to be injectable and have been used extensively for clinical cosmetic implants [194]. Different methods are used to fabricate these materials into a scaffold, such as spheroid formation, gas foaming, 3D printing, and electrospinning [195]. For liver regeneration, studies have shown that PLLA scaffolds support culture of primary hepatocytes prior to transplantation and can help maintain cell morphology and function [196]. PLLA can also be combined with various factors to support hepatocyte viability and function. One study demonstrated that 3D PLLA scaffolds supported fetal liver cell culture, and that combining scaffolds with the cytokine oncostatin M stimulated the maturation of hepatic parenchymal cells in culture, however they did not conjugate the oncostatin M directly to the PLLA scaffold [106]. Most studies report the use of PLA and PLLA in vitro*,* however there is evidence that they are also an effective cell vehicle for in vivo transplantation. One example combined PLA with gelatin and added bFGF to form scaffolds that promoted angiogenesis and hepatocyte survival and function after implantation into the mesentery of hepatectomized rats [105]. It is reported that injection of PLA and PLLA causes stimulation of macrophage-mediated inflammation leading to erythema, swelling and bruising, which should be a consideration in future transplantation applications [197].

#### Poly(Ɛ-caprolactone) (PCL)

Poly(Ɛ-caprolactone) (PCL) is an FDA-approved aliphatic polymer with a semi crystalline structure and a glass transition temperature of − 60 °C, giving softness and flexibility at body temperature [133]. PCL can be fabricated for liver regeneration into a variety of scaffold types such as nanofibers and 3D porous structures [198]. Although the ability to easily control pore size and fiber orientation during fabrication gives PCL and other synthetic materials an advantage over natural biomaterials, many researchers have turned to combinations of PCL and natural materials to promote superior cell viability and function. For example, Semnani et al.*,* combined PCL and chitosan into a nanfibrous scaffold by co-electrospinning of both materials. They were able to control pore size of the scaffold to promote mouse liver epithelial cell infiltration, PCL provided good mechanical properties to the scaffold, and the use of chitosan promoted cell adhesion and proliferation in vitro [199]. Another study combined PCL with natural decellularized ECM that was seeded with HepG2 cells treated with factors to promote ECM secretion [107]. They found that these hybrid scaffolds were able to support in vitro HepG2 survival and expression of liver-specific genes better than scaffolds without ECM deposition. Lastly, the biocomposite poly(L-lactic acid)-co-poly (Ɛ-caprolactone) (PLACL), formed from PLA and PCL, has been shown to promote hepatocyte differentiation from human MSCs when combined with natural materials such as collagen into a nanofibrous scaffold [200].

#### Poly (lactic-co-glycolic) acid (PLGA)

**Fig. 4:**
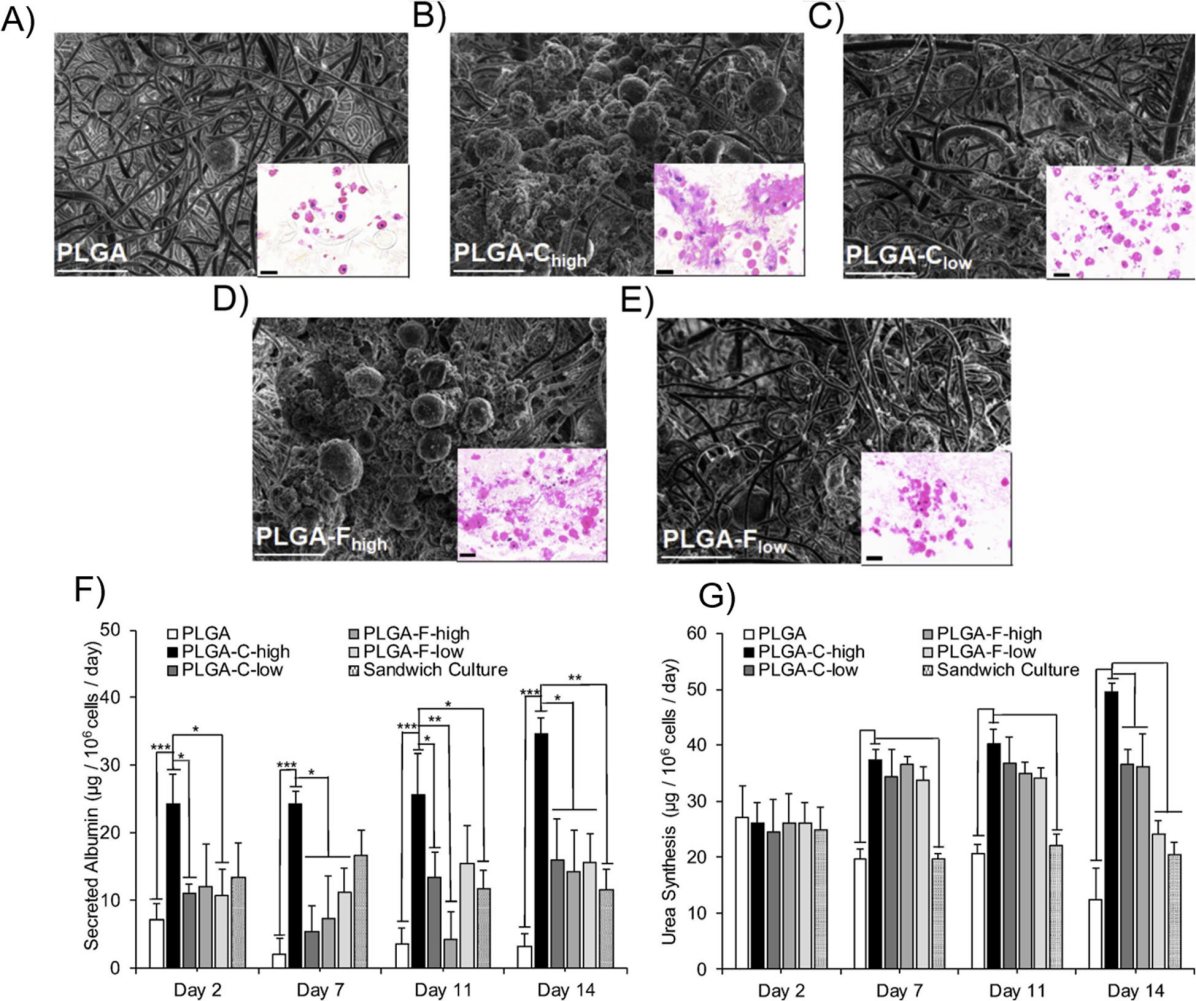
Function of primary hepatocytes in electrospun PLGA scaffolds is improved with the addition of collagen 1. Scanning electron micrographs (SEM) of primary human hepatocytes within electrospun PLGA scaffolds after 14 days of culture in (**a**) unmodified PLGA, **b** PLGA with high [collagen], **c** PLGA with low [collagen], **d** PLGA with high [fibronectin] and **e** PLGA with low [fibronectin], scale bar = 50 μm. Insets: representative H&E stained hepatocytes within scaffolds, scale bar = 20 μm. **f** In vitro albumin secretion by hepatocytes cultured in PLGA scaffolds with/without collagen/fibronectin compared to sandwich culture. **g** In vitro urea synthesis of hepatocytes cultured in PLGA scaffolds with/without. Adapted with permission from Ref [201]

Poly (lactic-co-glycolic) acid is synthesized by a combination of PLA and polyglycolic acid (PGA) and like other synthetic biomaterials, it offers a similar tunability and range of mechanical properties for liver regeneration. PLGA is often combined with natural materials to form a hybrid scaffold for culture of hepatocytes, demonstrating the versatility of this material. One study used PLGA to form a patterned scaffold that was either coated with collagen or filled in with a softer 3D collagen hydrogel, into which encapsulated rat primary hepatocytes were seeded [109]. After 10 days of culture cells in the collagen dispersed into 3D spaces which promoted better hepatocyte aggregation and albumin secretion than PLGA scaffolds coated with collagen. Another study directly compared the addition of fibronectin or type I collagen in a 3D nanofibrous PLGA scaffold for the culture of primary human hepatocytes, finding the best liver-specific function with the addition of type I collagen [201] (Fig. 4). As fabrication technologies such as 3D bioprinting continue to develop [202], researchers can take advantage of these methods to tailor synthetic-based scaffolds for hepatocyte culture and transplantation.

## Future recommendations

The field of liver regeneration has made significant progress in the last few decades, from the design of biomaterials to culture and support hepatocyte function in vitro to their use for cell transplantation strategies in vivo. While progress has been made, there remain many hurdles to overcome to push these regenerative strategies into clinical feasibility, including improving cell viability and differentiation, supporting liver-specific function of the transplanted cells, and recapitulating the native liver environment to support cell function.

### Improving cell viability and differentiation

As with any cell delivery strategy, cells need to be able to survive the delivery process and remain at the site of injury in the host environment. Due to the lack of blood supply, inflammation and fibrosis, the chronically-diseased liver is a hostile environment for cell survival. We can take advantage of biomaterials for cell delivery to override the unsuitable environment to support cell viability, however many studies which transplant cells into the liver do not quantify cell survival. This makes it difficult to assess the effectiveness of biomaterials at supporting post-transplantation cell survival. Furthermore, transplanted cells must either be delivered as hepatocytes or be able to differentiate into hepatocyte-like cells. One study tested the ability of various culture ratios of MSCs and hepatocytes cultured in PLGA scaffolds to promote differentiation of MSCs, finding that a ratio of 1:5 MSCs to hepatocytes resulted in the best differentiation of MSCs and survival of hepatocytes. They also transplanted cell-seeded scaffolds into injured mice and reported that scaffolds promoted restoration of liver function and reduced graft rejection [70].

To support long term cell survival and differentiation into the hepatocytic lineage post-transplantation, biomaterials can be combined with soluble factors. ESCs can be differentiated into hepatocyte-like cells by applying a series of soluble factors, which has been reported using a cocktail of factors absorbed to a polyethyleneimine nanoparticle scaffold [63]. In vivo studies using this strategy are lacking. Biomaterials can also be combined with heparin to allow for binding of growth factors such as VEGF, HGF, and FGF-2 to promote cell survival and differentiation [105, 187, 203]. Aside from molecular factors, biomaterials can be functionalized with ECM proteins such as collagen and fibronectin [130]. Hepatocarcinoma cells have a different aggregation pattern when they are cultured on a PEG scaffold with proteins, suggesting that these proteins are important in cellular functioning [204]. Future studies should consider the use of these protein or factor conjugation strategies to support cell survival and differentiation for in vivo liver regeneration.

### Recapitulating liver tissue structure and organization

One of the most important areas of liver regeneration that biomaterials can contribute to is reestablishment of the structure and organization of lobules. The lobule is a highly organized structure, both in terms of cell type, physical organization, and metabolically, that is difficult to restore by injection of a homogenous cell suspension. Future work with biomaterials should explore how we might mimic this organization for transplantation. There are many examples of cell-seeded scaffolds and organoids for use in vitro, but it is unknown if these constructs are suitable for the transplantation of cells. Moving forward, it is possible that multiple cell types (e.g., hepatocytes, non-paranchymal cells such as hepatic stellate cells, or endothelial cells) will need to be transplanted together, and biomaterials will need to be designed that can support both cell types [24]. Furthermore, metabolic zonation of hepatocytes is also important for maintenance of their function. McCarty et al., demonstrated this by creating controlled gradients of insulin, glucagon and 3-methylcholanthreme in a microfluidic device and culturing hepatocytes [205]. Could a strategy like this – which was designed to study drug metabolism – be translated into a viable transplantation construct?

### Supporting hepatocyte function

After successful delivery of cells to the liver, cells must then perform liver-specific functions to promote regeneration. The liver is a difficult organ to regenerate because the hepatocytes are responsible for many essential but distinct functions in the body. Current studies generally use the measure of albumin and urea as indicators that transplanted cells are mature and functional. However, there is little standardization of what is adequate levels for function. Furthermore, can the level of liver functionality obtained in animal studies be translated to humans? It is possible that slight improvements in liver function in animal studies which are deemed statistically insignificant may translate to improved quality or prolongment of life for a patient with liver failure. Lastly, how many hepatocytes need to be replaced to see improved liver function, and what is the best way to quantify this? How many cells for delivery does this translate to? These are all important questions that will need to be tackled to move cell delivery strategies forward. Biomaterials can help to address these questions by creating microenvironments to promote hepatocyte function.

One key aspect of improving hepatocyte function is maintaining cell polarity. An interesting biomaterial design which allows this is the sandwich approach, wherein cells are cultured between two layers of a biomaterials [131, 186, 201]. One study built upon this approach by using a triple layered hepatic tissue, sandwiching a sheet of hepatocytes between two sheets of endothelial cells to mimic liver lobules [206]. They reported that cells deposited ECM between the sheets, and polarity and function of hepatocytes was maintained. The researchers suggest that the construct could be transplanted in vivo but do not demonstrate this, which highlights a commonality among studies of currently being confined to in vitro applications. Other properties of biomaterials need to be optimized to increase hepatocyte function, for example fluid shear stress and the elastic modulus [207]. Fluid shear stress is an important mechanical factor that affects hepatocyte function and phenotype by triggering mechanosensitive gene expression [208]. Normal hepatocytes are exposed to shear stress via blood flow and scaffolds designed for long-term hepatocyte culture should include this feature. Microfluidic studies have demonstrated that albumin production and urea secretion are also influenced by shear stress. Shear stress also regulates the phenotype of liver sinusoidal endothelial cells by mechanotransduction, and in the case of chronic liver disease there is an increase in ECM deposition that will confer force on resident liver cells [209]. The addition of physiological shear stress to hepatocytes prior to transplantation may increase their function and phenotypic stability after delivery. Ideally, a biomaterial would allow hepatocytes to be cultured in vitro while exposed to shear stress and could then be transplanted in the same material into the liver. Incorporating features such as cell polarity and elasticity into the biomaterial will help to increase the functionality of delivered cells to address the outstanding questions in the field.

## Conclusions

Chronic liver disease and cirrhosis is a widespread and untreatable condition that leads to lifelong impairment and death. The scarcity of liver transplantation options requires the development of new strategies to attenuate disease progression and reestablish liver function by promoting regeneration. Cell transplantation is one such strategy but is hampered by the lack of a standardized cell source that can remain viable and retain liver-specific function following transplantation. Biomaterials are becoming an increasingly promising option to both culture cells for transplantation as well as to deliver cells and support their in vivo viability and long-term function. There is a wide variety of both natural and synthetic biomaterials that are now established as preclinical delivery vehicles with their own unique advantages and disadvantages for liver regeneration. Further work is needed to refine the use of these biomaterials and combine them with novel strategies that recapitulate liver organization and function in order to translate this work to clinical use.

## Data Availability

Not applicable.

## Abbreviations

2D: Two dimensional
3D: Three dimensional
BMP: Bone morphogenetic protein
CD44: Cluster of differentiation 44
EC: Endothelial cell
ECM: Extracellular matrix
EGF: Epithelial growth factor
EGFR: Epithelial growth factor receptor
EpCAM: Epithelial cellular adhesion molecule
ESC: Embryonic stem cell
FGF: Fibroblast growth factor
G-CSF: Granulocyte colony-stimulating factor
HA: Hyaluronic acid
HC: Hepatocyte
HepG2: Hepatoma G2
HGF: Hepatocyte growth factor
Hh: Hedgehog
hiPSC: Human induced pluripotent stem cell
HSC: Hepatic stem cell
HUVEC: Human umbilical vein endothelial cell
IFN-γ: Interferon-γ
IGF: Insulin growth factor
IL: Interleukin
KC: Kupffer cell
LGR5: Leucine-rich repeat-containing G-protein coupled receptor
LPS: Lipopolysaccharide
LSEC: Liver sinusoidal endothelial cell
LT-α: Lymphotoxin-α
MSC: Mesenchymal stem cell
MyD88: Myeloid differentiation primary response 88
OLT: Orthotopic liver transplantation
PCL: Poly(Ɛ-caprolactone)
PEGDA: Poly (ethylene glycol) diacrylate
PGA: Polyglycolic acid
PH: Partial hepatectomy
PLA: Polylactic acid
PLACL: Poly(L-lactic acid)-co-poly (Ɛ-caprolactone)
PLGA: Poly (lactic-co-glycolic acid)
PLLA: Poly-L-lactic acid
SCF: Stem cell factor
TGF-β: Transforming growth factor-β
TLR4: Toll-like receptor 4
TNFR1: Tumor necrosis factor receptor 1
TNF-α: Tumor necrosis factor-α
VEGF: Vascular endothelial growth factor
Wnt: Wingless-related integration site
